# Alternative Criteria to Address Major Flaws of Common Evaluation Metrics for Dose Distributions Through Characterization of Stereotactic Irradiation Plans for Brain Metastases Using the High-Definition Dynamic Radiosurgery Platform

**DOI:** 10.7759/cureus.95773

**Published:** 2025-10-30

**Authors:** Kazuhiro Ohtakara, Kojiro Suzuki

**Affiliations:** 1 Department of Radiation Oncology, Kainan Hospital Aichi Prefectural Welfare Federation of Agricultural Cooperatives, Yatomi, JPN; 2 Department of Radiology, Aichi Medical University, Nagakute, JPN

**Keywords:** average gradient distance (agd), brain metastases, conformity index (ci), gradient index (gi), heterogeneity index (hi), high definition dynamic radiosurgery (hdrs), irradiated isodose volume (iidv), plan evaluation metrics, stereotactic radiosurgery (srs), volumetric-modulated arc therapy (vmat)

## Abstract

Purpose

This study aimed to reveal major flaws in common evaluation metrics for dose distributions of stereotactic radiosurgery (SRS) for brain metastases (BMs) and to present alternative methods that are more objective and more relevant to treatment outcomes through characterization of dose distributions with volumetric-modulated arcs (VMA) by a simple optimization method using the High Definition Dynamic Radiosurgery (HDRS) platform (Elekta AB, Stockholm, Sweden).

Materials and methods

The subjects were 37 lesions with the gross tumor volume (GTV) ranging from 0.04 cc to 48.09 cc (median: 7.32 cc), which were treated as solitary lesions. The main constituents of the HDRS are the 5-mm leaf-width, 160-leaf collimator Agility^®^ (Elekta AB) and the planning system Monaco^®^ (Elekta AB). The VMA-based SRS plan for each GTV was optimized by applying only three cost functions with prioritizing the steepness of dose falloff outside the GTV boundary. The prescription dose was uniformly assigned to the GTV *D*_V - 0.01 cc_, minimum dose of GTV minus 0.01 cc (*D*_>95%_), for GTV >0.20 cc and the *D*_95%_ for the GTV ≤0.20 cc. An average gradient distance (AGD) was devised as a more quantitative evaluation measure of the dose gradient, which was defined as the difference between the radii of spheres equivalent to irradiated isodose volumes (IIDVs) of 50% and 100% of a reference dose. The AGDs were calculated from the isodose surfaces (IDSs) of the prescription dose and the GTV *D*_eIIV (equivalent IIDV)_, minimum dose of IIDV equivalent to GTV.

Results

The GTV dose heterogeneity and the dose 2 mm inside the GTV boundary were significantly correlated with the GTV, being the most inhomogeneous and highest, respectively, at the GTV of 1.71 cc. The dose 2 mm outside the GTV boundary and the AGDs were significantly correlated with the GTV, being steepest and shortest, respectively, at the GTV of 0.72 cc. All the minimum volumes outside the GTV, receiving the GTV *D*_eIIV_, the prescription dose, and 50% of the prescription dose, increased significantly with increasing the GTV. However, both the conformity and gradient indices (CIs, GIs) showed significantly better values with increasing the GTV, regardless of the reference doses. The CIs showed significantly better values as the GTV coverage by the reference IDS decreased: the *D*_V - 0.01 cc_ (median: *D*_99.88%_) to the *D*_98%_, whereas the GIs showed significantly better values as the GTV coverage by the reference IDS increased and further became over-covered. The interlesion differences in the CIs and the GIs varied substantially depending on the definitions.

Conclusions

In the VMA-based SRS with the HDRS for single BMs, the dose gradients outside and inside the GTV boundary were steepest at the GTVs of 0.72 cc and 1.71 cc, respectively, and correlated significantly with the GTV, peaking at the threshold volumes. As the GTV increased, both the CIs and GIs showed significantly better values, leading to less clinical significance of the differences in the values. The GIs also showed significantly better values as the target coverage by a reference IDS increased. The CI values ​​were significantly affected by the target coverage and the definition. In evaluating dose distributions of SRS for BMs, the absolute values such as the IIDVs should be prioritized over the common indices based on relative ratios.

## Introduction

Single or multi-fraction stereotactic radiosurgery (SRS) is an essential treatment option for brain metastases (BMs) [1,2]. Representative technological advances in SRS using standard general-purpose linacs include frameless six-degree-of-freedom image guidance with flexible dose fractionation, increased irradiation efficiency with flattening-filter-free (FFF) beams, and sophisticated dose distributions with volumetric-modulated arcs (VMA) [3]. VMA is the most suitable and indispensable technique for maximizing the effectiveness, safety, and efficiency of linac-based SRS, especially equipped with a 5-mm leaf-width multileaf collimator (MLC) and is the core technology of the High Definition Dynamic Radiosurgery (HDRS), the platform for SRS using linacs provided by Elekta AB (Stockholm, Sweden) [4-6]. The 5-mm leaf-width MLC Agility^®^ (Elekta AB) and the treatment planning system (TPS) Monaco^®^ (Elekta AB) form the basis of the HDRS [4,5]. The VMA using the HDRS without maximum dose constraints within a target volume (TV) maximizes the dose conformity to the TV boundary and the steepness of dose gradient outside the boundary by actively accepting internal ultra-high doses within the TV and substantial variations of the maximum doses [6,7]. Internal dose escalation within the gross tumor volume (GTV) can enhance tumor response after SRS [8,9]. In the HDRS, the VMA optimization can be achieved simply and efficiently by applying only three cost functions to the GTV alone, without using any dummy region of interest (ROI) [6,7].

There are various differences in the definition of a planning target volume (PTV), i.e., an isotropic margin size added to the GTV, the isodose surface (IDS) on which a prescription dose is specified, and the dose distribution among modalities and facilities [10,11]. In dose prescription to the PTV, the GTV marginal dose, which is the standard for conventional SRS, can vary substantially and is frequently unclear [12]. Accordingly, the local treatment outcomes, specifically the degrees of maximum response and adverse radiation effects (AREs), inevitably vary substantially between institutions, and it is currently difficult to reach a consensus on the optimal dose given the current adverse diversity of dose prescription methods [9]. In addition, currently the most prevailing dose prescription for an IDS that cover a specific and fixed percentage, e.g., 95-98%, of the TV, especially for the GTV, leads to a steady increase in the uncovered TV and may increase the likelihood of residual viable tumor at the tumor margin, as the GTV increases [10,12]. Therefore, highly objective evaluation criteria for the dose and quality of treatment plans are needed, regardless of intentions and preferences for dose prescription.

Since 2018, we have prioritized the boundary of GTV, rather than the PTV with ≥1 mm margin, as the standard surface for optimizing the dose distribution and have placed emphasis on maintaining the biological effective dose (BED) of the GTV boundary at a certain level or higher by using flexible dose fractionation of 3-15 times with sufficient consideration of volume effects [7,12,13]. Specifically, the prescription dose has been a BED of ≥80 Gy, based on the linear-quadratic formula, with the alpha/beta ratio of 10, BED_10_, which is assigned to the *D*_V - 0.01 cc_ to reduce the GTV below the dose to <0.01 cc, a stricter standard than <0.035 cc by the International Commission on Radiation Units and Measurements (ICRU) [10,12]. At the same time, we have ensured an appropriate dose attenuation margin at ≥2 mm outside the GTV boundary, specifically a BED_10_ of ≥50 Gy, fully considering various uncertainties relevant to irradiation accuracy and microscopic brain infiltration by tumors [14]. The appropriateness of the dose attenuation margins include avoiding too steep of a dose falloff such as BED_10_ <48 Gy for small GTVs and preferable dose escalation such as BED_10_ ≥60 Gy for large GTVs [14].

In this study, we first aimed to characterize the dose distributions in the VMA-based SRS for single BMs using the HDRS with the simple optimization method, especially its correlation with GTV. Through this, we revealed the major flaws of common evaluation metrics for dose distributions and presented alternative criteria that are more objective and more relevant to treatment outcomes. We particularly emphasized the low clinical significance of common metrics based on relative ratios such as conformity and gradient indices compared with absolute values such as an irradiated isodose volume (IIDV).

## Materials and methods

This study was conducted as part of a comprehensive study (approval number: 20240830-01) approved by the Clinical Research Review Board of Kainan Hospital Aichi Prefectural Welfare Federation of Agricultural Cooperatives.

The study subjects were 37 lesions in 28 patients who previously underwent multi-fraction SRS for BMs in our hospital, and some of which overlapped with the previous study subjects [6,7,12,14]. The GTV statistics were as follows: range: 0.04-48.09 cc; median value: 7.32 cc; and interquartile range (IQR): 7.71-20.40 cc. The treatment platform was the HDRS including the linac Infinity^®^ (Elekta AB) equipped with the MLC Agility^®^ and the TPS Monaco^®^ Version 5.51.10. A 6 MV flattening filter free beam was used with the maximum dose rate of 1400 monitor units/min [5,7]. The image co-registrations and the structure contouring prior to SRS planning were performed using the MIM Maestro^®^ Version 7.1.3 (MIM Software Inc., Cleveland, OH, USA) as described previously [12].

Each lesion was treated as a solitary metastasis, and a VMA-based SRS plan was created for each GTV using the simplest and the most suitable method at present based on previous planning studies [7]. The prescription dose was uniformly assigned to the GTV *D*_V - 0.01 cc_ (*D*_>95%_) for GTV >0.20 cc and the *D*_95%_ for GTV ≤0.20 cc [12]. This unconventional dose regimen was adopted to maintain a constant BED_10 _at the GTV boundary using flexible dose fractionation, regardless of the GTV, to attain excellent local control even for large lesions [7,13]. The irradiation isocenter was set at each GTV center, and the arc placement, collimator angle setting, and optimization methods were unified as described previously [6,7]. In brief, the only structures used for optimization were the GTV and body contour, without creating any dummy ROIs. Only three cost functions were applied to the two structures to prioritize dose conformity to the GTV boundary and the steepness of dose gradient outside the boundary, without any maximum dose constraints within the GTV [7]. The grid spacing and the statistical uncertainty of an X-ray voxel Monte Carlo algorithm were set as 1 mm and 1.00% per calculation, respectively [7]. After the optimization and dose calculation, the GTV coverage by the prescription dose was rescaled to the specified percentage [7]. For dose evaluation, additional structures were created by uniformly expanding and contracting the GTV boundary by 2 mm: GTV + 2 mm and GTV - 2 mm [7,15]. The GTV - 2 mm structures were created only for the GTVs of ≥0.496 cc (33 lesions) [15].

Common metrics and their alternatives used to assess the quality of SRS plans are summarized in Table 1 [16,17].

**Table 1:** Definitions of the metrics used for plan evaluation. *The abbreviations include those named uniquely in this study. **D_5%_ is applied for GTV ≤0.20 cc. ***D_95%_ is applied for GTV ≤0.20 cc. **** The rPCI is equivalent to the mPITV divided twice by the target coverage ratio (TV_PIV_/GTV). GTV: gross tumor volume; HI: heterogeneity index or homogeneity index; CI: conformity index; GI: gradient index; mRTOG: modified Radiation Therapy Oncology Group; *D*_0.01 cc_: minimum dose to 0.01 cc, receiving the near-maximum dose, of target volume (TV) (*D*_<5%_); *D*_V - 0.01 cc_: minimum dose of TV minus 0.01 cc (*D*_>95%_); ICRU: International Commission on Radiation Units and Measurements; *D*_X%_: minimum dose of X% of TV; TV_PIV_: TV within prescription isodose volume (PIV); IIDV: irradiated isodose volume; mPITV: modified ratio of PIV to TV; PITV: ratio of PIV to TV; mGI: modified GI; *D*_eIIV_: minimum dose of IIDV equivalent to TV.

Target volume	Category	Metrics (Abbreviation)*	Numerator	Denominator
GTV	HI	HI_mRTOG	*D*_0.01 cc_**	*D*_V - 0.01 cc_*** (prescription dose [PD])
		HI_ICRU	*D*_2%_ - *D*_98%_	*D*_50%_ (median dose)
	CI	Paddick's CI (PCI)	TV_PIV_ × TV_PIV_	GTV × PIV
		Reciprocal of PCI (rPCI)	GTV × PIV	TV_PIV_ × TV_PIV_
		mPITV****	PIV	GTV
		PITV_*D*_98%_	IIDV of *D*_98%_	GTV
	GI	mGI_*D*_eIIV_	IIDV of 50% of *D*_eIIV_	IIDV of *D*_eIIV_ (= GTV)
		mGI_*D*_98%_	IIDV of 50% of *D*_98%_	IIDV of *D*_98%_
		mGI_*D*_V - 0.01 cc_ (= original GI)	IIDV of 50% of *D*_V - 0.01 cc_ (50% PIV)***	IIDV of D_V - 0.01 cc_ (= PIV)
GTV + 2 mm	GI	mGI_*D*_eIIV_	IIDV of 50% of *D*_eIIV_	IIDV of *D*_eIIV_

These include those that are currently in common use and those that were alternatively defined to reveal their flaws.

Additional and complementary parameters used for plan evaluation are summarized in Table 2.

**Table 2:** Additional dosimetric parameters used for plan evaluation. *D_95%_ is applied for GTV ≤0.20 cc. **D_5%_ is applied for GTV ≤0.20 cc. ***If the value is too low, e.g., < 71.2% or the biologically effective dose < 48 Gy with the alpha/beta ratio of 10, the coverage of microscopic brain infiltration by tumor and uncertainty in irradiation accuracy may be reduced. GTV: gross tumor volume; PD: prescription dose; *D*_near-max_: near maximum dose; AGD: average gradient distance; IDS: isodose surface; IIDV: irradiated isodose volume.

Evaluation items	Metrics	Definitions	Interpretation
GTV dose heterogeneity	PD to *D*_near-max_	*D*_V - 0.01 cc_* (%) relative to *D*_0.01 cc_** (100%)	The lower the value, the more heterogeneous
Steepness of dose increase inside GTV boundary	GTV – 2 mm *D*_eIIV_ to GTV *D*_eIIV_	GTV – 2 mm *D*_eIIV_ (%) relative to GTV *D*_eIIV_ (100%)	The higher the value, the steeper the dose increase inside the GTV boundary
GTV dose conformity	GTV coverage with *D*_eIIV_	GTV coverage (%) with GTV *D*_eIIV_	The closer to 100%, the better the conformity
	IIDV spillage of GTV *D*_eIIV_	GTV *D*_eIIV_ IIDV minus GTV (cc)	The smaller the volume, the better the conformity
	PIV spillage	PIV minus GTV (cc)	The smaller the volume, the better the conformity
Appropriateness of dose attenuation margin	GTV + 2 mm *D*_eIIV_ to GTV *D*_eIIV_	GTV + 2 mm *D*_eIIV_ (%) relative to GTV *D*_eIIV_ (100%)	The lower the value, the steeper the dose falloff outside the GTV boundary***
Steepness of dose gradient outside the GTV	50% PIV spillage	IIDV of 50% of PD minus GTV (cc)	The smaller the volume, the steeper the dose gradient outside the GTV boundary
	AGD from prescription IDS	Difference (mm) between radii of spheres equivalent to IIDVs of 50% and 100% of PD	The shorter the distance, the steeper the dose gradient outside the prescription IDS
	AGD from *D*_eIIV_ IDS	Difference (mm) between radii of spheres equivalent to IIDVs of 50% and 100% of GTV *D*_eIIV_	The shorter the distance, the steeper the dose gradient outside the GTV boundary

The original dose heterogeneity index (HI) defined by the Radiation Therapy Oncology Group (RTOG) is the ratio of the maximum dose (*D*_max_) divided by the minimum dose (*D*_min_) of a GTV, which corresponds to those displayed generally on a dose-volume histogram (DVH), based on the smallest voxel unit, e.g., 0.001 cc [15]. However, dose prescription to the GTV *D*_min_ is currently rarely adopted, and a *D*_0.001 cc_ as the *D*_max_ has little practical clinical significance, given the uncertainties of irradiation accuracy in frameless irradiation [12,15]. The GTV *D*_min_ is also more susceptible to small differences in GTV delineation. In addition, the differences between the *D*_max_ and the *D*_2%_ and between the *D*_min_ and the *D*_98%_ steadily increase with increasing the GTV. Accordingly, the differences between the *D*_2%_ and the *D*_98%_ of the GTV definitely underestimate the inherent GTV dose heterogeneity more as the GTV increases [14]. In SRS targeting even small GTVs, dose evaluation in 0.01 cc units was deemed appropriate: *D*_V - 0.01 cc_ as the near-minimum dose (*D*_near-min_) and *D*_0.01 cc_ as the near-maximum dose (*D*_near-max_) for a GTV of >0.20 cc [12]. Therefore, the ratio of *D*_0.01 cc_ to *D*_V - 0.01 cc_ was defined as the HI_mRTOG and compared with the standard HI defined by the ICRU (Table 1). However, for the GTV of <0.20 cc, in which 0.01 cc is >5% of the GTV, the *D*_95%_ and the *D*_5%_ were applied as the *D*_near-min_ and the *D*_near-max_, respectively. In this study, the *D*_eIIV (equivalent irradiated isodose volume__)_, the minimum dose of the IIDV equivalent to a TV, was adopted as an objectively assessable marginal dose of the TV, regardless of the intended prescription dose [14,15]. Specifically, the *D*_eIIVs_ of the GTV, the GTV - 2 mm, and the GTV + 2 mm were evaluated.

Four differently defined conformity indices (CIs) were compared (Table 1). The most commonly used Paddick’s CI (PCI), its reciprocal (rPCI), and the RTOG CI were compared for the prescription IDS [18-22]. In addition, the RTOG CI was also calculated using the *D*_98%_, recommended by the ICRU [10]. The PITV value by the RTOG when the *D*_eIIV_ is used as the reference dose shows the perfect value of 1. The PCI or rPCI reflects the TV coverage by the *D*_eIIV_, adjusting the false perfection of the dose conformity. Four differently defined gradient indices (GIs) were calculated for the *D*_eIIV_, *D*_98%_, and *D*_V - 0.01 cc_ of the GTV and the *D*_eIIV_ of the GTV + 2 mm, considering the actual diversity of dose prescription methods [23,24]. In this study, an average gradient distance (AGD) was devised as a more quantitative evaluation measure than the conventional GI with a simple relative ratio. The AGD from the prescription IDS was defined as the difference between the radii of spheres equivalent to the IIDVs of 50% and 100% of prescription dose. Similarly, the AGD from the IDS of the GTV *D*_eIIV_ was calculated as the difference between the radii of spheres equivalent to the IIDVs of 50% and 100% of the GTV *D*_eIIV_.

Statistical analyses are based on paired nonparametric tests and were performed using the BellCurve for Excel® (version 4.05; Social Survey Research Information Co., Ltd., Tokyo, Japan). The distributions of numerical variables are shown as box-and-whisker plots (BWPs). The whiskers display the nearest values ≤1.5 times the IQR, and the cross marks beyond the lines show the outliers >1.5 times the IQR. Two numerical variables were compared using the Wilcoxon signed-rank test (WSRT). The correlation between two numerical variables was evaluated using Spearman’s rank correlation coefficient (SRCC). The Jonckheere-Terpstra (JT) test was used to assess trends of increase or decrease between three or more numerical variables. Statistical significance was considered at p <0.05 and expressed on a three-level scale: p <0.05 (*), p <0.01 (**), and p <0.001 (***). Significant p-values are marked in blue in the figures.

## Results

The GTV coverage by the *D*_V - 0.01 cc_ for the 34 GTVs of >0.20 cc ranged from 97.01% to 99.98% (median 99.88%), while the median GTV coverage value of all the lesions was 99.86%. The descriptive statistics of GTV *D*_98%_ relative to the prescription dose were as follows: range: 98.44-108.01%; median value: 105.60%; and IQR: 103.38-106.25%. The GTV *D*_98%_ significantly increased as the GTV increased (SRCC: rho = 0.814, p <0.001 ***).

**Figure 1:**
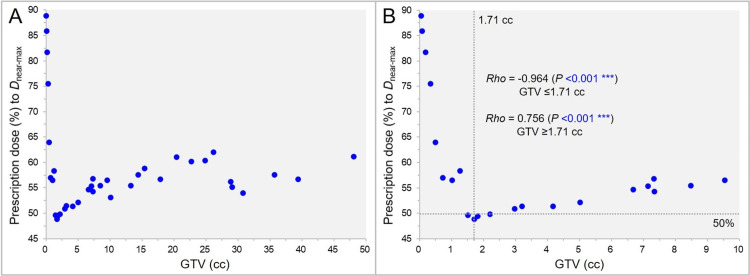
The gross tumor volume (GTV) dose heterogeneity as a function of GTV. The scatter plots (A,B) show the correlation between the GTV and the prescription isodose (%) relative to the near-maximum dose (*D*_near-max_, 100%); with the GTV up to 50.00 cc (A) and 10.00 cc (B), along with the results of Spearman’s rank correlation coefficient (SRCC) divided by the GTV of 1.71 cc (B). The dotted lines in B indicate the GTV of 1.71 cc, with the GTV dose being the most inhomogeneous, and the 50% isodose.

The correlation between the GTV and the dose heterogeneity is show in Figure 1.

The GTV dose was the most inhomogeneous with the 48.9% IDS covering at the GTV of 1.71 cc that corresponds to a sphere with a diameter of 14.8 mm, while it was the most homogeneous at the smallest GTV of 0.04 cc with the 88.9% IDS covering (Figure 1B). The GTV dose heterogeneity correlated significantly with the GTV: positively for the GTV ≤1.71 cc; albeit negatively for the GTV ≥1.71 cc (Figure 1A, 1B).

**Figure 2:**
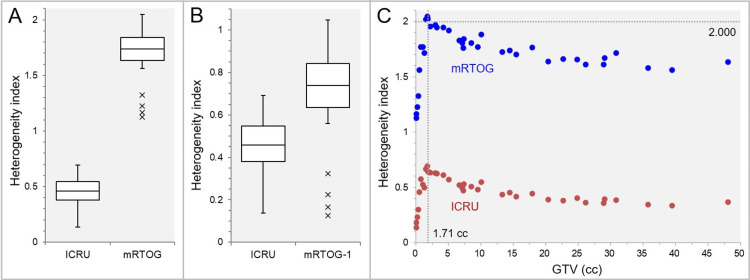
Comparison of two differently defined indices in the evaluation of gross tumor volume (GTV) dose heterogeneity as a function of GTV. The box-and-whisker plots (BWPs) (A,B) show the comparisons of two heterogeneity indices (HIs): the ICRU vs. the mRTOG (A); and the ICRU vs. the mRTOG minus 1 (mRTOG-1) (B). The scatter plot (C) displays the correlation between the GTV and the two HIs. The dotted lines in C indicate the GTV of 1.71 cc and the HI value of 2.000. The results of Spearman’s rank correlation coefficient (SRCC) divided by the GTV of 1.71 cc are omitted because they are similar to those in Figure 1. ICRU: International Commission on Radiation Units and Measurements; mRTOG: modified Radiation Therapy Oncology Group.

The distributions of the two HIs, the HI_ICRU and the HI_mRTOG in Table 1, are shown in Figure 2A, 2B.

The HI_ICRU values were <1 and significantly smaller than those of the HI_mRTOG (Figure 2A). To make it easier to compare, when the HI_mRTOG was subtracted by 1, the range and IQR of the HI_ICRU were clearly smaller than those of the HI_mRTOG (Figure 2B). The correlations between the GTV and the two HIs are shown in Figure 2C. The interlesion differences in the values of GTV dose heterogeneity were clearly smaller in the HI_ICRU than in the HI_mRTOG (Figure 2C).

**Figure 3:**
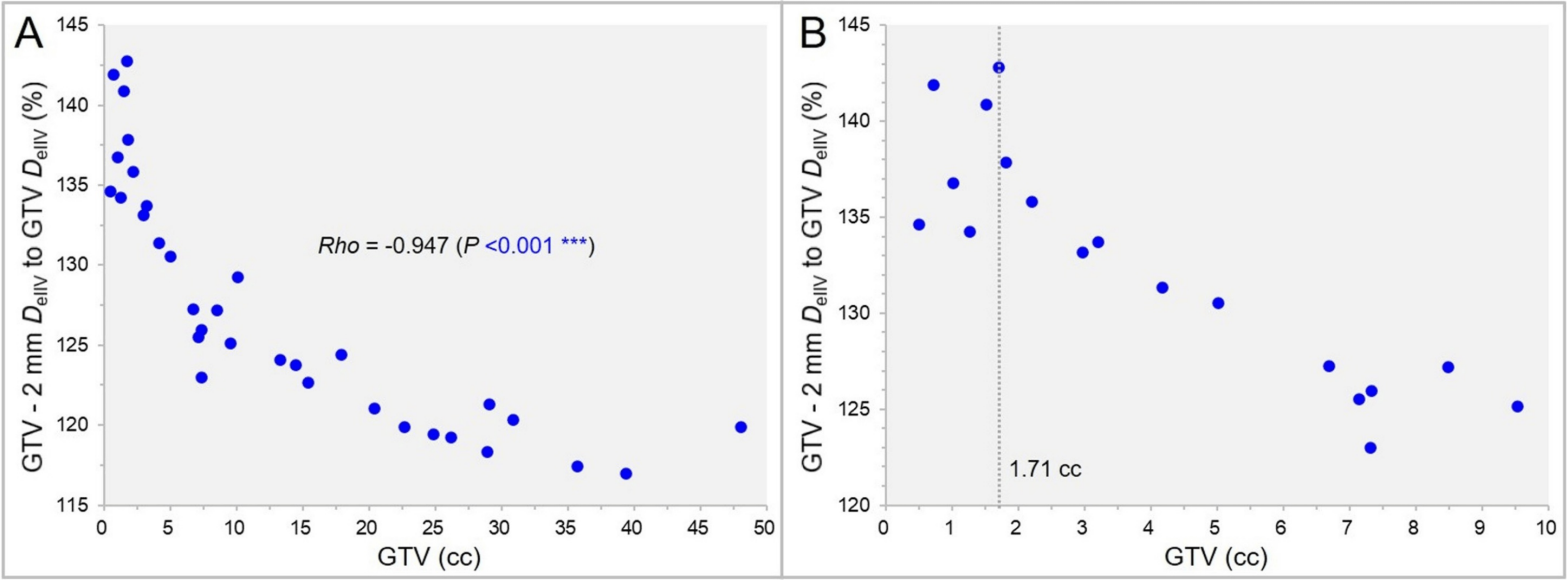
The dose at 2 mm inside the gross tumor volume (GTV) boundary as a function of GTV. The scatter plots (A,B) show the correlation between the GTV and the GTV – 2 mm *D*_eIIV_ (%) relative to the GTV *D*_eIIV_ (100%); with the GTV up to 50.00 cc (A) and 10.00 cc (B), along with the result of Spearman’s rank correlation coefficient (SRCC) (B). The dotted line in B denotes the GTV of 1.71 cc, with the GTV – 2 mm *D*_eIIV_ being the highest. *D*_eIIV_: minimum dose of irradiated isodose volume (IIDV) equivalent to a target volume (TV).

The correlation between the GTV and the GTV - 2 mm *D*_eIIV_ (%) relative to the GTV *D*_eIIV_ (100%) is shown in Figure 3.

The dose 2 mm inside the GTV boundary was significantly inversely correlated with the GTV, and was highest at the GTV of 1.71 cc (Figure 3A, 3B).

**Figure 4:**
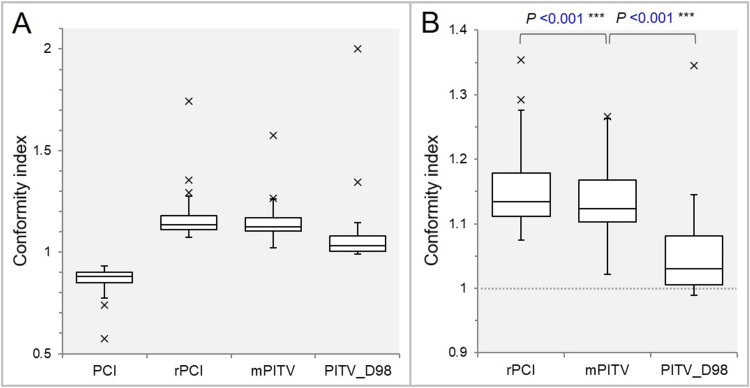
Comparisons of four differently defined indices for evaluating the gross tumor volume (GTV) dose conformity. The box-and-whisker plots (BWPs) (A,B) show the comparison of four conformity indices (A); and the comparison of three indices, excluding one largest outlier in each, along with the results of the Wilcoxon signed-rank test (WSRT) (B). The dotted line in B indicates the perfect value of 1.000. PCI: Paddick’s conformity index (CI); rPCI: reciprocal of PCI; mPITV: modified ratio of prescription isodose volume to target volume (GTV); PITV_D98: PITV by GTV *D*_98%_.

The distributions of the four CIs (Table 1) are shown in Figure 4A.

Whether the GTV or part of it was in the numerator or denominator determines whether the CI value was greater than or less than 1 (Figure 4A). The PCI values were <1 and smaller than its reciprocal (the rPCI), and the interlesion difference was also small compared with that of the rPCI, as the PCI applies large values, ​​including the prescription isodose volume (PIV), to the denominator (Figure 4A). The CIs for the GTV of 0.04 cc were the most deviant outliers in all the definitions (Figure 4A). When comparing without the PCI, the CIs decreased significantly in the order of the rPCI, the mPITV, and the PITV_D98% (JT test: p <0.001***) (Figure 4B). The interlesion difference was larger in the rPCI than in the mPITV, as the rPCI corresponds to the mPITV divided by the GTV coverage ratio of <1, inevitably increasing the rPCI values (Figure 4B). The PITV_D98%, which had lower GTV coverage values compared with those of the rPCI and the mPITV, showed the closest value to 1, i.e., the most excellent value (Figure 4B).

**Figure 5:**
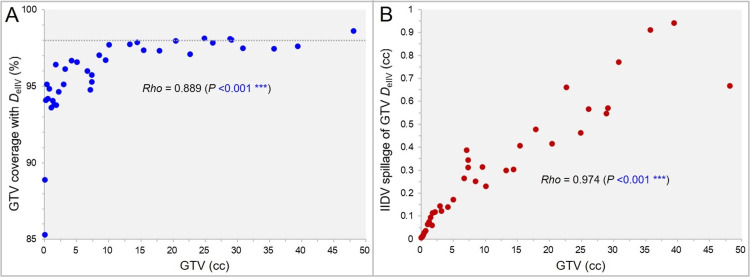
The metrics relevant to the gross tumor volume (GTV) DeIIV as a function of GTV. The scatter plots (A,B) show the correlations between the GTV and the GTV coverage value by the *D*_eIIV_ (A) and between the GTV and the IIDV spillage outside the GTV by the GTV *D*_eIIV_ (B), along with the results of Spearman’s rank correlation coefficient (SRCC) (A,B). The dotted line in A indicates 98% coverage of the GTV. *D*_eIIV_: minimum dose of irradiated isodose volume (IIDV) equivalent to a target volume (TV)

The correlation between the GTV and the GTV coverage value with the *D*_eIIV_ is shown in Figure 5A.

Overall, 29.7% and 89.2% of the cases had the GTV coverage of <95% and <98%, respectively, by the *D*_eIIV_ (Figure 5A). The GTV coverage value by the *D*_eIIV_ increased significantly with increasing the GTV (Figure 5A). The correlation between the GTV and the IIDV spillage of the GTV *D*_eIIV_, outside the GTV, is shown in Figure 5B. The IIDV spillage of the GTV *D*_eIIV_ increased significantly as the GTV increased (Figure 5B).

**Figure 6:**
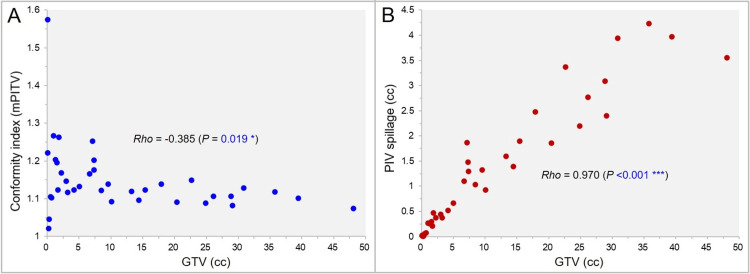
The conformity index (CI) by mPITV and the prescription isodose volume (PIV) spillage as a function of gross tumor volume (GTV). The scatter plots (A,B) show the correlations between the GTV and the CI value by mPITV (A); and between the GTV and the PIV spillage volume outside the GTV (B), along with the results of Spearman’s rank correlation coefficient (SRCC) (A,B). mPITV: modified ratio of prescription isodose volume to target volume (GTV)

The correlations between the GTV and the mPITV, the representative CI, and between the GTV and the PIV spillage outside the GTV are shown in Figure 6A, 6B.

The mPITV value decreased significantly, i.e., the GTV dose conformity appeared to be better, with increasing the GTV (Figure 6A). However, the PIV spillage outside the GTV increased significantly, similar to the IIDV spillage of the GTV *D*_eIIV_, as the GTV increased (Figure 6B).

**Figure 7:**
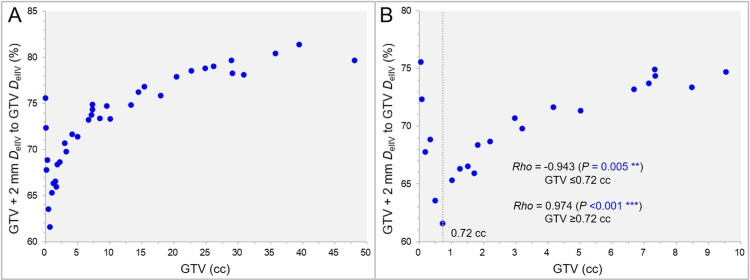
The dose attenuation margin at 2 mm outward from the gross tumor volume (GTV) boundary as a function of GTV. The scatter plots (A,B) show the correlation between the GTV and the GTV + 2 mm *D*_eIIV_ (%) relative to the GTV *D*_eIIV_ (100%) (A,B); with the GTV up to 50 cc (A) and 10 cc (B), along with the results of Spearman’s rank correlation coefficient (SRCC) divided by the GTV of 0.72 cc (A). The dotted line in B indicates the GTV of 0.72 cc at which the dose attenuation is the steepest 2 mm.

The correlation between the GTV and the GTV + 2 mm *D*_eIIV_ (%) relative to the GTV *D*_eIIV_ (100%) is shown in Figure 7.

The GTV + 2 mm *D*_eIIV_ was lowest, i.e., the dose gradient just outside the GTV boundary or the dose attenuation margin was steepest, at the GTV of 0.72 cc that corresponds to a sphere with a diameter of 11.1 mm (Figure 7A, 7B).

The GTV + 2 mm *D*_eIIV_ correlated significantly with the GTV: negatively for the GTV ≤0.72 cc; albeit positively for the GTV ≥0.72 cc (Figure 7A, 7B).

**Figure 8:**
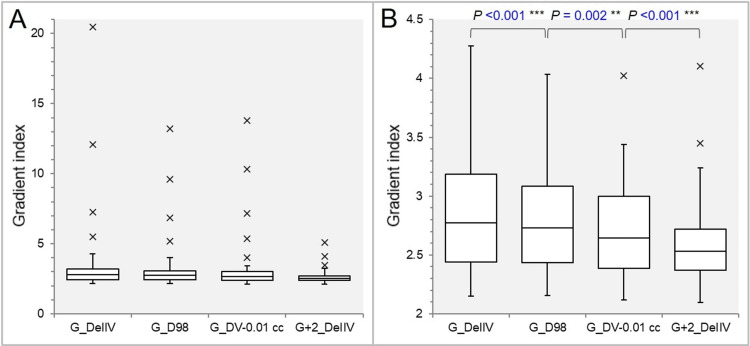
Comparison of four differently defined indices for evaluating the dose gradient outside the gross tumor volume (GTV). The box-and-whisker plots (BWPs) (A,B) show the comparison of four gradient indices (A); and the same comparison, excluding the outliers above 4.500, along with the results of the Wilcoxon signed-rank test (WSRT) (B). G_DeIIV: gradient index (GI) for the GTV *D*_eIIV_; G_D98: GI for the GTV *D*_98%_; G_DV-0.01 cc: GI for the GTV *D*_V-0.01 cc_; G+2_DeIIV: GI for the GTV + 2 mm *D*_eIIV_.

Figure 8 shows the distributions of the four differently defined GIs.

In particular, the four GIs for the GTV were the outliers with extremely high values of >5 ​​in the four smallest GTVs of ≤0.33 cc (Figure 8A). Therefore, the distributions shown in Figure 8B exclude the outliers of >4.5 for ease of comparison. The GIs decreased significantly in the order of the mGI_DeIIV for the GTV, the mGI_D98%, the mGI_DV-0.01 cc, and the mGI_DeIIV for the GTV + 2 mm (JT test: p = 0.008**) (Figure 8A, 8B). That is, the higher the GTV coverage with the reference dose, including the prescription dose, the lower the GI value was.

**Figure 9:**
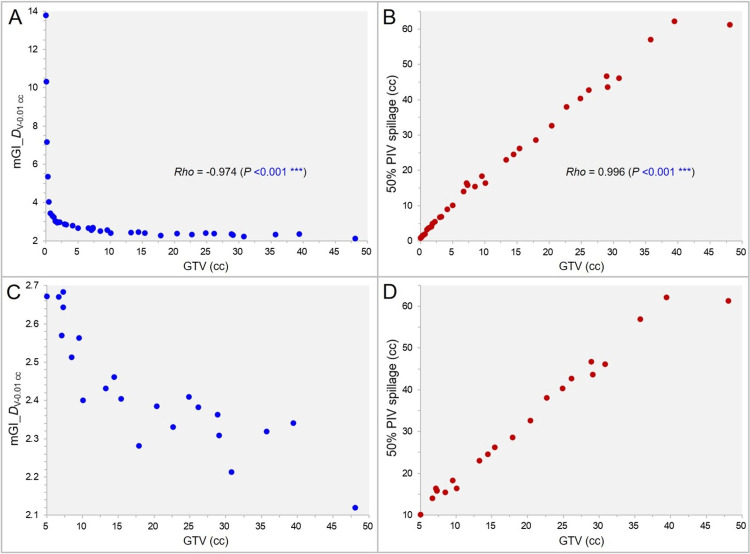
The gradient index (GI) and the 50% prescription isodose volume (PIV) spillage as a function of gross tumor volume (GTV). The scatter plots (A-D) show the correlations between the GTV and the GI value by the GTV *D*_V-0.01 cc_ (A,C) and between the GTV and the 50% PIV spillage volume outside the GTV (B,D); with the GTV of 0.00-50.00 cc (A,B) and 5.00-50.00 cc (C,D), along with the results of Spearman’s rank correlation coefficient (SRCC) (A,B). mGI_DV-0.01 cc: modified GI by the GTV *D*_V-0.01 cc_.

The correlations between the GTV and the mGI_DV-0.01 cc and between the GTV and the 50% PIV spillage are shown in Figure 9.

The mGI_DV - 0.01 cc decreased significantly, i.e., the dose gradient outside the GTV appeared to be better, with increasing the GTV (Figure 9A, 9C). However, the 50% PIV spillage increased significantly as the GTV increased (Figure 9B, 9D). The correlations between the other three GIs and the GTV were similar (data not shown).

**Figure 10:**
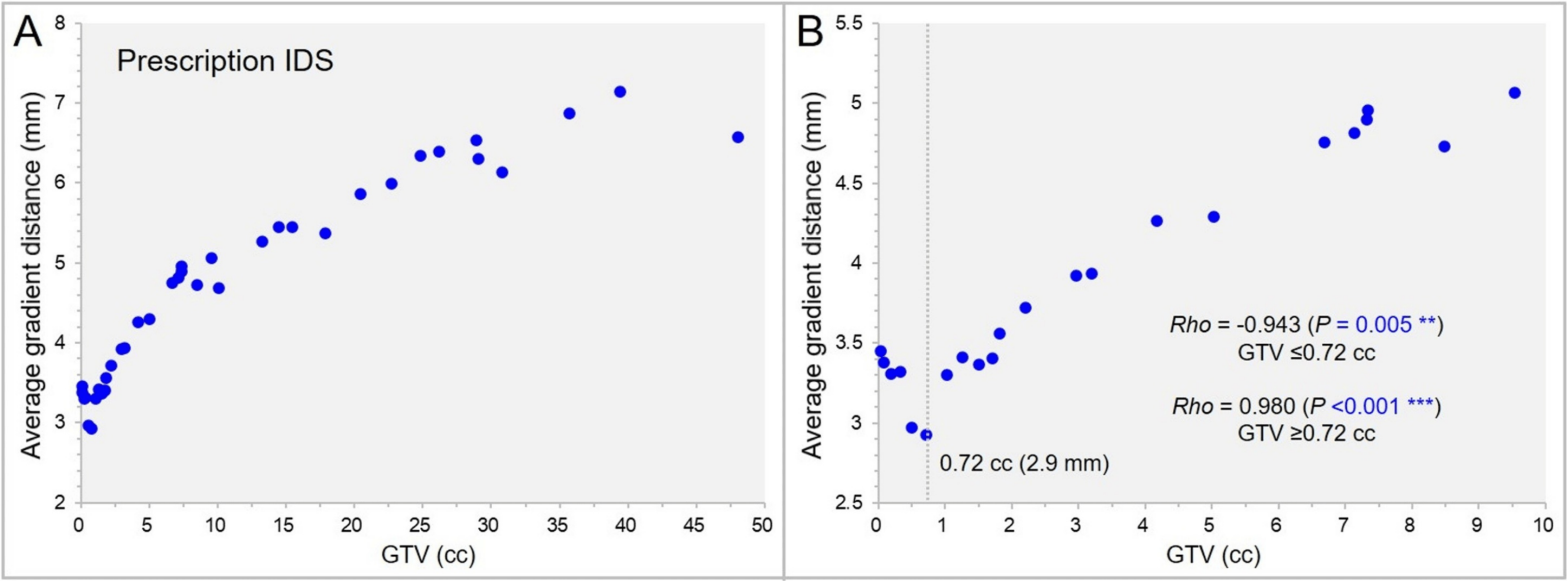
The average gradient distance (AGD) from the prescription isodose surface (IDS) as a function of gross tumor volume (GTV). The scatter plots (A,B) show the correlation between the GTV and the AGD from the prescription IDS; limited to GTV up to 10 cc in B, along with the results of Spearman’s rank correlation coefficient (SRCC) divided by the GTV of 0.72 cc (A). The dotted line in B denotes the GTV of 0.72 cc for which the AGD is the shortest at 2.9 mm.

Figure 10 shows the correlation between the GTV and the AGD from the prescription IDS, the GTV *D*_V - 0.01 cc_ for the GTV >0.20 cc.

The descriptive statistics of the AGD from the prescription IDS were as follows: range: 2.9-7.1 mm; median value: 4.8 mm; and IQR: 3.5-5.9 mm. The AGD from the prescription IDS was shortest, i.e., the dose gradient was steepest, at the GTV of 0.72 cc (Figure 10A, 10B). The AGD from the prescription IDS correlated significantly with the GTV: negatively for the GTV ≤0.72 cc; albeit positively for the GTV ≥0.72 cc (Figure 7A, 7B).

**Figure 11:**
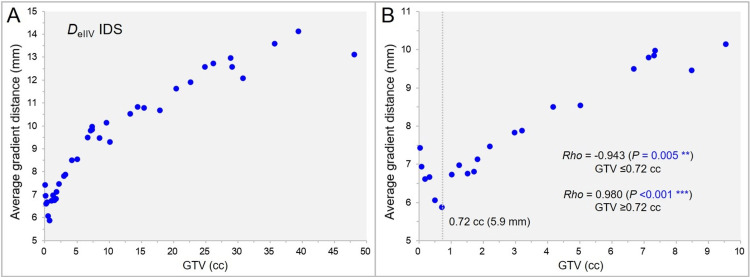
The average gradient distance (AGD) from the isodose surface (IDS) of gross tumor volume (GTV) DeIIV as a function of GTV. The scatter plots (A,B) show the correlation between the GTV and the AGD from the IDS of GTV *D*_eIIV_; limited to GTV up to 10 cc in B, along with the results of Spearman’s rank correlation coefficient (SRCC) divided by the GTV of 0.72 cc (A). The dotted line in B denotes the GTV of 0.72 cc for which the AGD is the shortest at 5.9 mm. DeIIV: minimum dose of irradiated isodose volume (IIDV) equivalent to a target volume (TV)

Figure 11 shows the correlation between the GTV and the AGD from the IDS of the GTV *D*_eIIV_.

The statistics of the AGD from the IDS of GTV *D*_eIIV_ were as follows: range: 5.9-14.1 mm; median value: 9.5 mm; and IQR: 7.1-11.6 mm. The AGD from the IDS of GTV DeIIV was significantly longer than that from the prescription IDS (WSRT: p <0.001 ***). The AGD from the IDS of GTV DeIIV was also shortest at the GTV of 0.72 cc and showed a similar correlation with the GTV as the AGD from the prescription IDS (Figure 10).

## Discussion

In the dose distributions of VMA-based SRS for single BMs using the HDRS by the simple optimization method prioritizing the steepness of dose falloff outside the GTV, the dose gradients inside and outside the GTV boundary were steepest at the GTVs of 1.71 cc and 0.72 cc, respectively, and correlated significantly with the GTV, peaking at the threshold volumes. Although the steep dose increase inside the GTV boundary is basically advantageous for enhancing the anti-tumor efficacy, it may increase the risk of tumor swelling and/or intratumoral bleeding, especially with single high-dose irradiation [8,9,15]. Furthermore, in fractionated SRS, significant tumor shrinkage during irradiation inevitably increases the surrounding brain dose, increasing the risk of AREs [15,25]. In particular, for SRS of ≥5 fractions or for treatment periods of one week or longer, imaging evaluation is recommended approximately once a week [25]. When irradiating multiple lesions simultaneously, the greater the number of lesions and the closer they are to each other, the less steep the dose gradients inside and outside the GTV boundary become and the more likely they are to vary due to dose interference. It is also necessary to adjust excessively steep dose attenuation margins in small lesions, especially for tumors with highly invasive tendency [14].

Currently, the most common dose reporting for SRS of BMs is limited to two points: the marginal dose, the definition of which varies substantially among facilities and modalities, and the maximum or central dose [10,11]. The recommendations of the ICRU report 91 include the *D*_98%_, *D*_50%_, and *D*_2%_ for dose evaluation of a TV, especially for ≥2 cc [10]. These doses may be reasonable as representative values ​​of the DVH curve and for homogeneous target doses. However, these dosimetric criteria are deemed inappropriate for SRS of BMs, which is characterized by steep dose gradients inside and outside the target boundary [12]. It is also essential to clarify the GTV marginal dose, irrespective of dose prescription policy, to establish and reach consensus on the optimal doses of SRS for BMs [12]. In addition, it is also recommended to clarify the dose 2 mm outside the GTV boundary [14]. The dose 1 mm outside the GTV may be estimated as the intermediate value of the marginal doses of the GTV and the GTV + 2 mm. Each *D*_eIIV_ can be an objective representative of these marginal doses.

In evaluating the target dose heterogeneity, the HI_mRTOG with a minimum volume unit of 0.01 cc for GTV ≥0.20 cc is more appropriate compared with the original PITV by the RTOG (0.001 cc unit), the HI_ICRU (2% unit), and the *D*_2%_ divided by the *D*_98%_ (2% unit). The GTV *D*_V - 0.01 cc_ (%), relative to the *D*_0.01 cc_ (100%), or its reciprocal is sufficient to objectively indicate the dose heterogeneity for GTVs of ≥0.20 cc. Converting this percentage into a ratio naturally reduces the differences in the numbers themselves among lesions and plans and has little clinical significance. In practice, the dose 2-4 mm inside the GTV boundary was assumed to be more relevant to clinical outcomes such as a nadir tumor response, compared with the *D*_near-max_ or the *D*_50-80%_; however, this needs to be further verified [15]. For the GTV of ≥1.71 cc, the dose 2 mm inside the GTV boundary significantly decreased with increasing the GTV, which was deemed advantageous in our dose prescription policy [12,14,15]. The number of dose fractions is flexibly increased as the GTV increases, extending the treatment period. Decreasing the steepness of the internal dose increase with increasing the GTV may reduce the risk of AREs relevant to significant tumor shrinkage. The GTV dose heterogeneities varied substantially in the VMA-based SRS with the HDRS. In conventional forward planning for dynamic conformal arcs, it is common to determine the target dose heterogeneity, e.g., 70% IDS covering, in advance and plan based on that. Maintaining a constant dose inhomogeneity is of little clinical significance and is physically limited for various tumor volumes, and the steepness of dose gradient outside the GTV should be prioritized, especially in inverse planning.

This study revealed the major flaws in evaluating using CIs and GIs: first, the larger the GTV, the better these values [24]. The clinical significance and impact of a difference in the index value, e.g., a difference of 0.10, vary greatly depending on the GTV and definitely decrease as the GTV increases. For larger GTVs, comparisons should be made using stricter criteria such as the IIDVs excluding the GTV [26,27]. On the other hand, for smaller GTVs, both the CI and GI values ​​naturally deteriorate, and the adequate target coverage should be prioritized. The second flaw is that these indices are significantly affected by the target coverage value with a reference IDS [22,24]. Even in the same case, the GI value is better when the target coverage by a reference IDS is higher. Even if the prescription dose is the same, the GI value is better when prescribed to the PTV periphery rather than the GTV boundary, and the larger the PTV margin is. The CI values ​​are better with insufficient target coverage, e.g., *D*_98%_, than with sufficient coverage, e.g., *D*_V - 0.01 cc_ [22]. Compromising the GTV coverage to improve the CI values ​​is inappropriate as it may lead to insufficient local tumor control. Thus, the major flaws of CI and GI values ​​are that they are based on relative ratios, so superior values does not necessarily correlate with superior treatment outcomes [28,29]. Therefore, more quantitative and objective evaluation criteria based on absolute values should be prioritized over these relative ratio-based indices, irrespective of dose prescription and planning policy. For dose conformity, the IIDV spillage by the GTV *D*_eIIV_ reliably reflects the increased normal tissue high-dose radiation exposure with increasing the GTV. For dose gradients outside the GTV, the AGD (mm) from the IDS of the GTV *D*_eIIV_ can quantitatively demonstrate the effects of radiation exposure on normal tissue just outside the GTV more clearly than GIs, although it is difficult to calculate in practice. Taken together, the use of ratio-based metrics should be avoided in evaluating the dose distributions, and priority should be given to the IIDVs relevant to AREs [26,27]. IIDVs can be easily calculated and compared from the DVH. Comparison of multiple candidate plans can be easily performed using the DVH of the ROI with a sufficient margin of at least 1 cm around the GTV.

Knowledge regarding the tolerable doses of the normal brain is not yet mature, and there are two accuracy issues: IIDV calculation and diagnosis of brain radionecrosis [27]. Accurately drawing the brain contour is actually complicated, and cerebrospinal fluid cavities and/or bones are frequently included in the IIDVs [30]. Studies that strictly exclude structures other than brain tissue for dose evaluation are rather rare [30]. Furthermore, because it is difficult to distinguish regrowth of residual tumor at the margin of the lesion from brain radionecrosis on imaging, many cases diagnosed as brain radionecrosis contain viable tissue. There is a need to review and further accumulate more accurate knowledge regarding dose-volume factors, including high doses more relevant to brain radionecrosis [26].

Study limitation

The number of lesions studied, 37, may be insufficient. The reproducibility of the 1.71 cc and 0.72 cc threshold volumes needs to be confirmed in a larger number of subjects with various localizations and shapes. The applied optimization method for the VMA cannot be determined to be the best in terms of dose gradient steepness, and it may be possible to further improve it by combining different cost functions and/or different settings of the relevant parameters. Many facilities still prefer less inhomogeneous GTV doses, e.g., ≥80% IDS covering, for SRS of BMs. The clinical superiority of the extremely inhomogeneous GTV doses adopted in this study needs to be clarified in the future. It should also be noted that dose distributions as actual absorbed doses in intracranial SRS are dynamic and difficult to predict due to blurring caused by intra-fractional head movement in frameless irradiation and possible changes in the shape and/or displacement of the lesion during fractionated irradiation [25].

## Conclusions

In SRS with VMA by the simple optimization method using the HDRS for single BMs, the dose gradients inside and outside the GTV boundary were steepest at the GTVs of 1.71 cc and 0.72 cc, respectively, and correlated significantly with the GTV, peaking at the threshold volumes.

As the GTV increased, both the CIs and GIs showed significantly better values, leading to less clinical significance of the differences in the values. The GIs also showed significantly better values as the target coverage by a reference IDS increased. The CI values ​​were significantly affected by the target coverage and the definition. The interlesion differences in the HIs, CIs, and GIs varied substantially depending on the definitions. In evaluating dose distributions of SRS for BMs, the absolute values such as the IIDVs should be prioritized over the common indices based on relative ratios.
